# A case of inflammatory mass resulting from calcium crystal deposition disease around the thoracic spine

**DOI:** 10.1016/j.radcr.2022.09.040

**Published:** 2022-10-04

**Authors:** Kaori Mizutomi, Yasuji Ryu, Natsuki Sugimori, Toshiyuki Okamura, Hiroyuki Hayashi, Hiroshi Kawai

**Affiliations:** a Department of Radiology, Tonami General Hospital, Toyama, Japan; b Department of Internal Medicine, Tonami General Hospital, Toyama, Japan; c Department of Orthopedics, Tonami General Hospital, Toyama, Japan

**Keywords:** Computed tomography, Magnetic resonance imaging, Calcium crystal deposition diseases, Calcification, Calcium pyrophosphate dihydrate, Acute back pain

## Abstract

Calcium crystal deposition diseases are transient benign diseases that can cause intense pain. They can sometimes cause masses and soft tissue edema around the calcification, which should be differentiated from tumors and abscesses. We report a case of calcium crystal deposition disease with an enhanced mass on the ventral side of the vertebral bodies resembling tumors and abscesses.

A female patient in her 50s visited our hospital complaining of chest pain. Computed tomography revealed a soft tissue mass with polygonal high-density lesions on the ventral side of the thoracic spine. Initially, we suspected it to be a perivertebral tumor and considered a biopsy. However, the pain rapidly improved with the administration of oral acetaminophen (Caronal, Chuo-ku/Tokyo/Japan). Hence, the patient was followed up for the time being. The mass disappeared after 3 months. In addition, polygonal high-density lesions inside the mass disappeared over time. Therefore, it was diagnosed as an inflammatory mass due to calcium crystal deposition disease.

Calcium crystal deposition diseases can cause soft tissue edema and inflammatory mass around the calcium crystal deposit that can be confused with a perivertebral tumor. This report elucidates the importance of identifying calcifications within and near the masses to diagnose an inflammatory mass resulting from calcium crystal deposition.

## Clinical presentation

The patient was a 50-year-old woman with no medical history and a chief complaint of sudden chest and back pain during the night. The following day, the patient had persistent esophageal pain and opted to consult a family doctor. The cause of the pain was unknown, and the patient was referred to our hospital for evaluation. The pain was the strongest 3 days after onset and had improved slightly when the patient visited our hospital 5 days later. Upon examination, vital signs were normal, and the pain was exacerbated by physical activity. Routine blood test results showed a mildly increased inflammatory response (C-reactive protein level, 1.99 mg/dL [normal value is less than 0.15 mg/dL]).

## Investigation/Imaging findings

**Fig. 1:**
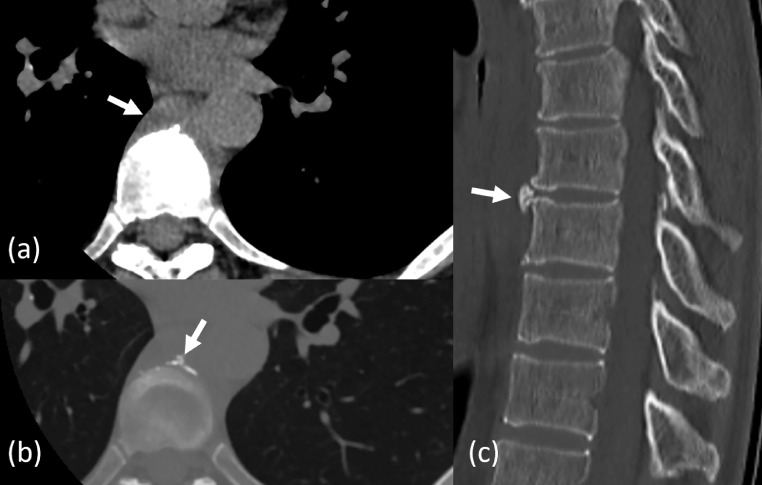
Computed tomography images of the Th6-9 vertebral bodies. (a) The axial view shows a soft tissue mass (arrow) extending ventral to the Th6-9 vertebral bodies. (b) The axial and (c) sagittal views of the bone condition reveal a polygonal high-density lesion (arrow) on the dorsal side of the mass and the ventral side of the Th8-9 vertebral bodies, respectively.

Computed tomography(CT)scan showed a 70 mm × 8 mm soft tissue mass extending ventral to the Th6-9 vertebral bodies in the craniocaudal direction (Fig. 1). The lesions showed uniform mild high density (20-30 Hounsfield Unit), and no low-density areas suspected of simple fluid were observed. A polygonal high-density lesion was observed on the dorsal side in the soft tissue mass and the ventral side of the Th8-9 vertebral bodies, although the clinical importance was unknown at the time.

**Fig. 2:**
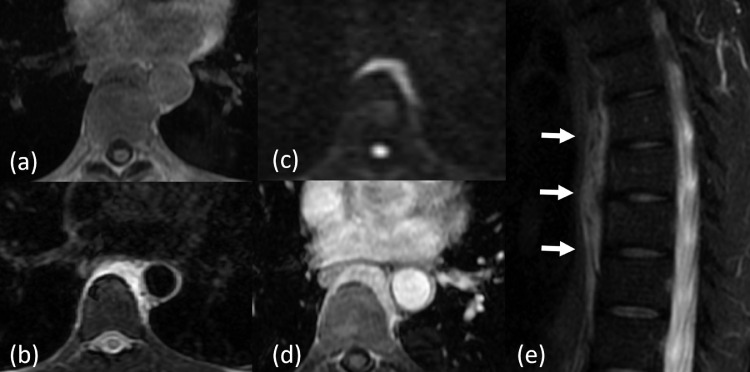
Magnetic resonance images of the thoracic spine. (a) The mass shows low signal intensity of the T1-weighed gradient-echo image. (b) Fat suppressed T2-weighted image revealing high signal intensity. (c) Diffusion-weighted image showing diffusion limitation. (d and e) Gadolinium-enhanced dynamic magnetic resonance imaging reveals the overall delayed contrast enhancement of the mass (arrows).

The next day, magnetic resonance imaging (MRI) showed the lesion with low signal intensity on the T1-weighted gradient-echo image and high signal intensity on the fat-suppressed T2-weighted image (Fig. 2). Diffusion-weighted imaging showed diffusion limitation. Gadolinium-enhanced dynamic MRI showed an overall delayed contrast enhancement. There was no MRI signal on the ventral side of the Th8-9 vertebral bodies; hence, it was suspected to be a calcium crystal deposit or ossification. There were no abnormal signals in the bones or intervertebral discs. Since the lesion was enhanced overall, it was unlikely to be an abscess or edema and was considered a soft tissue tumor.

## Differential diagnosis

Hematological disease, metastatic tumor, immunoglobulin 4 (IgG4)-related disease, fibroma, neurogenic tumor, hemangioma, and esophageal lesion were considered in the differential diagnosis. If the Th8/9 ventral high absorption is considered to be calcification in the mass rather than osteophyte, we also had to differentiate diseases such as tumoral calcinosis, gout, and calcium crystal deposition diseases.

## Treatment/outcome/follow-up

**Fig. 3:**
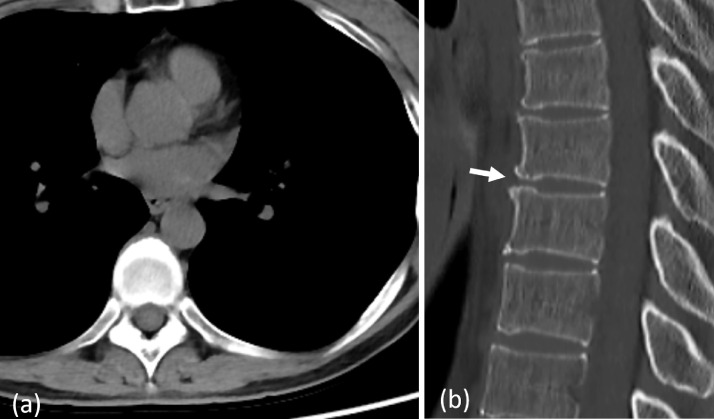
Computed tomography images of the thoracic spine 4 months later. (a) The mass is not observed. (b) The high density on the ventral side of the Th8-9 vertebral bodies are not visible (arrows).

Additional blood test results showed no abnormalities in antinuclear antibodies, antineutrophil cytoplasmic antibodies, soluble interleukin-2 receptor, serum IgG4, and negative tumor markers (carcinoembryonic antigen, carbohydrate antigen 19-9, squamous cell carcinoma antigen, neuron-specific enolase, and cytokeratin 19 fragment). Upper gastrointestinal endoscopy showed no abnormalities. A posterior mediastinum mass was suspected, and a biopsy was considered. However, the pain significantly improved with the oral administration of acetaminophen; therefore, the patient was followed up. The patient was to be re-examined after 3 months and a biopsy was to be performed if the soft tissue mass had grown. MRI 3 months later showed that the mass had disappeared completely, and CT 4 months later remained clear with no sign of the mass (Fig. 3). In addition, the high density on the ventral side of the Th8-9 vertebral bodies had disappeared, and it proved to be a transient calcification. We considered this case to be a metabolic disease with calcium crystal deposition of the intervertebral disc's anterior longitudinal ligament or annulus fibrosus. Furthermore, we diagnosed the mass on the ventral side of the vertebral bodies as an inflammatory reaction caused by a metabolic disease.

## Discussion

We experienced a case of a rapidly painful calcified mass on the ventral thoracic spine that resolved spontaneously. It was undoubtedly determined to be metabolically active calcium deposits with its resulting mass. Massive particular calcinosis of the soft tissue mass is not rare, and various diseases are known. Among them, tumoral calcinosis, gout, and calcium crystal deposition diseases can produce metabolically active calcifications.

Tumoral calcinosis is a rare familial condition characterized by painless, periarticular masses [1,2]. This disease has characteristic cystic or lobulated calcifications inside the tumor, and especially, within the cystic calcifications, it is accompanied by fluid-calcium levels, termed the *sedimentation sign* [2]. In our case, tumoral calcinosis was ruled out because the calcifications were neither lobed, nor cystic, nor were there fluid-fluid levels caused by calcium layering. Moreover, there was no family history of this disease.

Gout is a metabolic disease that can result from sustained hyperuricemia and form calcified masses. Gout can cause focal erosions of the underlying bone, and when the mass is large, calcification may appear [2]. A diagnosis of gout was discarded in this case because patient was female, had almost no alcohol history, and had no history or family history of gout. Additionally, there was no bone erosion around the mass.

Calcium crystal deposition diseases include hydroxyapatite deposition disease and calcium pyrophosphate dehydrate (CPPD) deposition disease. Calcium crystal deposition diseases are acute and transient benign lesions, and our case followed a very similar clinical course. Hydroxyapatite deposition disease occurs mostly around joints, especially tendons. The localization of the calcification in our case was thought to be the anterior longitudinal ligament or the annulus fibrosus, and there was no tendon on the ventral side of the thoracic vertebrae, suggesting that hydroxyapatite deposition disease was unlikely. Comprehensively, the mass on the ventral side of the vertebral body was considered to be an inflammatory mass due to CPPD deposition disease.

CPPD deposition disease, often occurring in the limb joints, also referred to as pseudogout, is not necessarily symptomatic if the crystals are localized. However, when the crystal mass collapses and is absorbed by the joint cavity or tissues with blood circulation, an inflammatory or a local abnormal immune reaction via polynuclear leukocytes that phagocytose the crystal occurs [3].

Acute spinal CPPD deposition disease is rare compared to joint development. In acute spinal CPPD deposition disease, an inflammatory mass may form around the calcium crystal deposit as the inflammation spreads. Various sites where the mass may form, such as intervertebral discs, posterior vertebral bodies, and spinous processes, have been reported, although they often have a contrast enhancement and need to be differentiated from vertebral discitis, epidural abscess, and metastasis [4], [5], [6].

Acute CPPD deposition disease in the cervical spine is widely recognized as crowned dens syndrome and is often treated appropriately [5,6]. Conversely, if it occurs in a lesion other than in the cervical spine, it may be misdiagnosed as septic vertebral discitis and treated unnecessarily with antibiotics [6].

In this case, the entire mass show contrast enhancement. In general, edema does not show contrast enhancement, and abscesses are contrast-enhanced only on the margin, and the inside is liquid. Therefore, the mass was neither edema nor an abscess. Moreover, our mass disappeared, including a calcium crystal. It was presumed to be an inflammatory mass caused by a mechanism similar to that in CPPD deposition disease.

This case was considered a calcium crystal deposition disease in the anterior longitudinal ligament or annulus fibrosus of the intervertebral disc. Although anterior longitudinal ligament calcification is reportedly very rare [7], calcification of the disc, including the annulus fibrosus, is common. In our case, the mass formed on the ventral side of the Th8-9 vertebral bodies that covered this calcium crystal deposit, and it was necessary to distinguish it from osteophytes. Therefore, the imaging uncertainty of this case was whether the CT high-density lesion found inside the mass was an osteophyte or calcium crystal deposit.

**Fig. 4:**
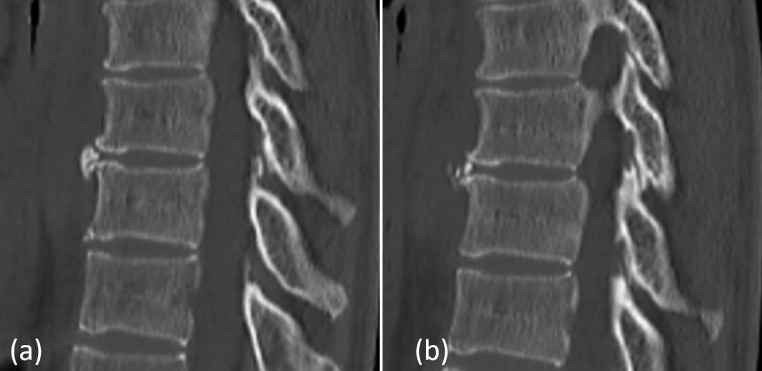
Initial computed tomography findings. (a) The polygonal high-density lesion appears continuous from the joint surface, and it is difficult to distinguish between ossification and calcification. (b) There is high absorption of fine particles around the polygonal obstruction.

Ossification differs from calcification in that osteoblasts and osteoclasts are found within and are extremely common, as osteophytes exist around the vertebral bodies. Nishikawa et al. [8] compared the image features of calcification of the cervical ligamentum flavum and ossification of the ligamentum flavum. Ossification of the ligamentum flavum shows a beak-like morphology consistent with the articular surface, whereas the calcification of the ligamentum flavum of the cervical region shows oval with pale speckled morphology. In this case, the polygonal lesion appeared to be continuous from the joint surface, and it was difficult to distinguish between ossification and calcification. However, when observed in detail, there was high absorption of fine particles around it, suggestive of calcification (Fig. 4).

Li et al. [9] reported calcification of the C3-4 and C4-5 discs and the C3-4 posterior longitudinal ligament in a 7-year-old girl. In their case, no abnormal signal was observed around the calcified area, and the pre-vertebral soft tissue of C2-6 showed swelling and gadolinium enhancement. These calcifications and masses were confirmed to disappear over time. This suggests that an inflammatory mass can form even if not in contact with the calcification site.

We experienced calcium crystal deposition disease, probably CPPD deposition, forming an inflammatory mass. In calcium crystal deposition disease, an inflammatory mass may form around the calcium crystal deposit. Calcium crystal deposition disease is benign and can be expected to improve spontaneously. Therefore, it is crucial to consider this disease to avoid unnecessary biopsies and antibiotic treatments. If a high-density lesion is found inside or near the mass, calcium crystal deposition disease should be considered.

## Patient consent

Written informed consent was obtained from the patient to publish this case report, including accompanying images.
